# Surface Preparation and its Effect on Sticking

**DOI:** 10.1007/s11095-026-04052-0

**Published:** 2026-04-02

**Authors:** Henrietta Tsosie, James Thomas, John Strong, Antonios Zavaliangos

**Affiliations:** 1 Drexel University Materials Science and Engineering Department, 3141 Chestnut St. LeBow 344, Philadelphia, PA 19104 USA; 2 Global Pharmaceutical Sciences, AbbVie, Inc., North Chicago, IL 60064 USA

**Keywords:** punch surface chemistry, solvent cleaning, sticking, wettability, x-ray photoelectron spectroscopy

## Abstract

**Purpose:**

This study examined how controlled punch surface preparation and subsequent atmospheric aging influence adhesion between punch metal and formulation powder during tablet compaction. We aimed to clarify how the evolving surface state of S7 tool steel affects sticking for representative excipients and active pharmaceutical ingredients (APIs), and to establish a reproducible approach for surface state control in material-sparing sticking studies.

**Methods:**

Removable S7 tool steel punch tips were mechanically polished, chemically cleaned, and rinsed to create a defined “as-cleaned” surface. Punches were aged at 55% RH for 10 min to 72 h. Sticking of mannitol, Starch 1500, acetylsalicylic acid (ASA), and ibuprofen (IBU) was quantified using a non-contact laser reflectivity sensor and measuring adhered mass after single compactions. Static contact angle measurements tracked surface evolution, and X-ray photoelectron spectroscopy (XPS) characterized chemical changes.

**Results:**

Freshly cleaned punches showed strong adhesion for mannitol and starch. Starch lost its sticking tendency within minutes of aging, while mannitol remained adhesive until several hours of exposure. ASA and IBU exhibited moderate sticking that declined slightly with aging. Contact angle and XPS indicated rapid formation of a carbonaceous/oxide overlayer, reducing polar surface energy and altering adhesion pathways.

**Conclusions:**

Punch surface chemistry evolves quickly under ambient conditions, strongly impacting sticking behavior. Cleaning to testing time can alter adhesion outcomes within minutes, highlighting the need to standardize surface preparation and define the punch-surface state. This framework offers a practical basis for surface control in pharmaceutical compaction studies.

## Introduction

Powder adhesion to tooling, or *sticking*, is a common issue in tableting operations. Few formulation components, typically (but not exclusively) the active pharmaceutical ingredient (API), may detach from the tablet and remain on the tools after ejection. Accumulation of the adhered material during production results in a progressively worsening tablet surface quality, loss of geometry definition, as well as increased weight variation of the tablet. When present, sticking may result in significant costs and production delays. Despite repeated attempts to establish causal relationships between material and process conditions and the development of sticking, a fundamental understanding of the problem is still elusive. This situation makes mitigation difficult and requires extensive experimental investigations. Recent work has focused attention on the assessment of sticking risk using material sparing techniques [1] and the use of a laser reflectivity sensor [2], an adhesive punch [3], and a removable punch [4].

Although not always openly reported, there have been concerns for the excessive variability in the test results [5] and unexplainable changes of sticking behavior results without a detectable change in the material properties or tableting process [6]. Some of these issues were initially attributed to the measuring technique itself [5]. Recently, our group developed a non-contact laser sensor that measures the reflection of a laser beam from the punch surface. Reduced reflection compared to an unused punch corresponds to the partial coverage of the punch surface by sticking materials [2]. While this non-contact sensor has a simple operation and high sensitivity, during validation studies, we became aware of variability induced by surface preparation, which motivated us to understand the role of the state of the surface of the punch on sticking.

Guided by common practice in surface science experiments, we present here a consistent surface punch preparation procedure for sticking studies. The process involves material removal, and targeted cleaning with successive rinses. We used simple wettability measurements with a consistently low contact angle as quality control of the surface preparation. We also present aging effects for cleaned punches which can be as rapid as 1–10 min of exposure to the atmosphere. The effect of punch aging varied among the tested materials and, in some cases, dramatically changed the sticking behavior of the punch with the same excipient. These findings highlight the need to carefully control the consistency of punch preparation and even the time between cleaning and sticking experiments. These results improve the quality of the sticking experiments especially when small quantities of material are available and augment our mechanistic understanding for this phenomenon.

Material sparing techniques for quantifying sticking have received considerable attention recently. They often require sensitive measurements of the adhered mass at levels below 1 mg. These techniques rely on mass measurements or quantification of area covered by the adhered material. High performance liquid chromatography (HPLC) [2, 7–11], mass measurements using a removable punch tip [4, 12–16], tablet-tooling separation forces [3, 17], scanning electron microscopy (SEM) [18], and a non-contact laser surface apparatus [2] have been used for this purpose. Some of the advantages and disadvantages of these techniques are listed in Table I.

**Table I:** Current Early Detection Techniques Available

Method	Mass adhered (mg)	Advantages	Disadvantages	References
High performance liquid chromatography	< 0.001	Quantitative, high accuracy, sensitivity and specificity	Requires removal, compaction run interruption, requires special equipment, laborious	[9]
Removable punch tip	0.01	Quantitative, can be combined with other techniques, simplicity	Requires removal, compaction run interruption, no chemical identification or morphological information	[4]
In-line laser	0.001–1	Non-contact, in-line measurement, sensitive, quick, simplicity	No chemical identification or morphology information, saturation at full coverage while mass accumulates, usable only for early stages of sticking	[2]
Scanning electron microscopy	N/A	Provides morphology and identification, area coverage	Requires removal of punch tip, electron beam interaction, requires special equipment, laborious	[18]

All evaluation techniques require some basic cleaning of the punch, at least to remove deposits from handling. In addition, if the punch had been previously used, traces of any material that remained on it must also be removed. Previous studies of sticking have paid insufficient attention to surface preparation. The term “thorough cleaning” has been often used but typically involved only a single solvent (e.g., isopropyl alcohol) and no proof of consistency of the cleaning process was provided [3, 12, 17]. Unfortunately, visual inspection and “mirror finish” of the surface does not guarantee the absence of residual materials on the punch or any level of consistency of the punch surface chemistry. The visual detection of contaminants on highly reflective metal surfaces (such as polished punch faces) depends on the contaminant’s size relative to the wavelength of visible light (λ = 400–700 nm) and its optical contrast with the reflecting background. Particles smaller than the visible wavelengths and/or with small refractive index differences with the background surface make scattering very weak and therefore any visual detection very challenging [19]. In this case, a hazy appearance develops at specific angles only when a high level of area coverage by the contaminant exists. In addition, optical aberrations, scattering from eye structures, or limitations in retinal resolution and their variation in vision from person-to-person [20] make visual inspection completely unreliable for assessing the cleanliness of the surface.

**Fig. 1:**
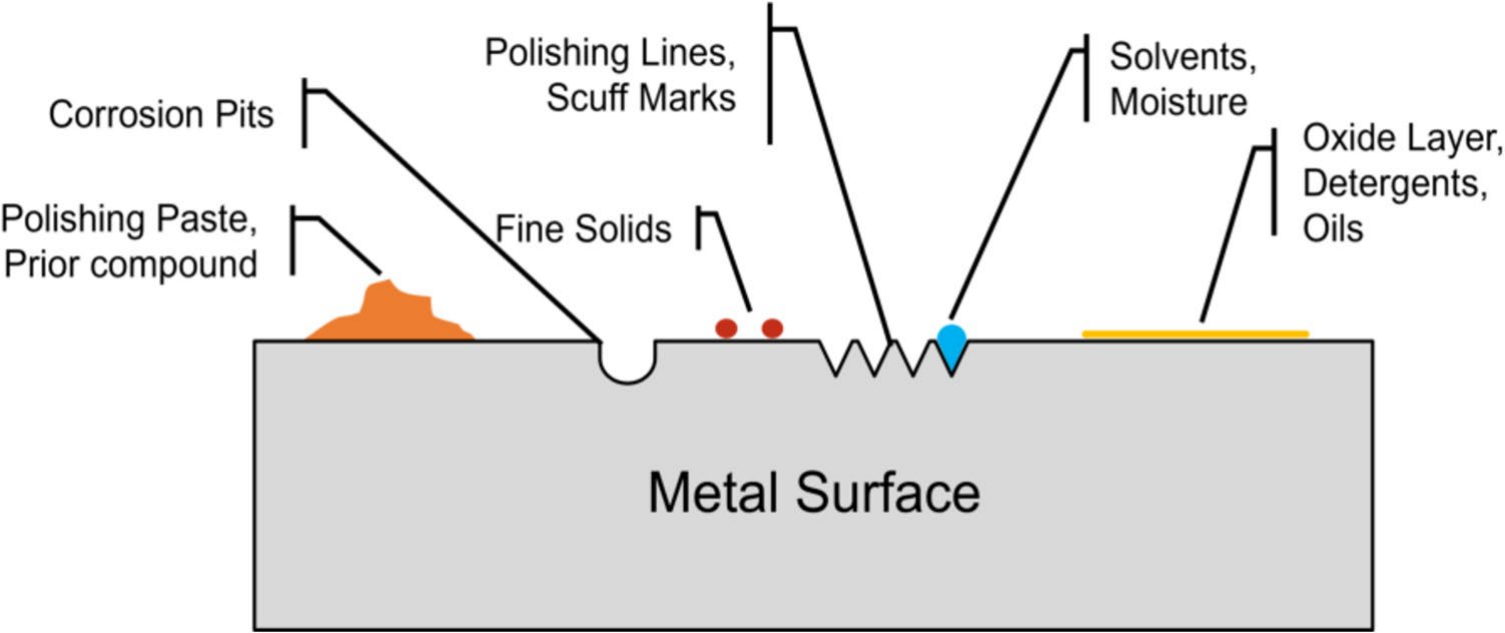
Typical steel contaminants.

A more systematic approach should explicitly consider the various sources and types of potential contamination. Compaction punch surfaces possess a certain degree of roughness and may contain bulk particle deposits of multiple origins, along with retained oils, moisture, solvents, and corrosion byproducts, Fig. 1. Even in the absence of external deposits, there exists a layer on the order of 2–10 nm in which the surface composition is different from the composition of the bulk material due to adsorption or oxidation [21, 22]. If multiple species are present on the surface, different steps may be required to remove them.

To address these challenges, we drew on surface preparation techniques developed for applications where contamination control is critical, such as atomic force microscopy, electronics manufacturing, and ultra-high vacuum systems [23–25]. These techniques consist of a multistep process involving both mechanical and chemical removal of surface contaminants. Mechanical polishing can remove any strongly adhered or difficult to dissolve deposits on the surface and is achieved by successive steps of polishing depending on the amount of material to be removed with rinsing steps between each polish to flush away remaining debris or polishing medium. Removing residues from the polishing medium requires specific solvent rinses and clean glass containers to avoid cross-contamination. Depending on the residues present from previous polishing steps, a series of rinses and ultrasonication in polar and non-polar solvents are conducted. Such solvents include acetone, ethanol, methanol and water (distilled or deionized) [26]. For drying between solvent steps, a dry inert gas (e.g., nitrogen or argon) can be used. In addition to solvent cleaning, detergents containing surfactants, followed by a rinsing step are used to remove both hydrophilic and hydrophobic chemical groups. Their usage also reduces the ability of these contaminants to redeposit on the surface after removal [25, 27].

Mechanical polishing removes the topmost surface layer, exposing a fresh metal surface that readily interacts with the environment. This surface has a higher surface energy and can immediately react with oxygen, carbon dioxide, and organic compounds in the atmosphere, leading to changes in wettability across many materials [22, 24, 28, 29]. In the polymer coatings industry, the presence of contamination is known to lead to decohesion [30], but little has been reported regarding powder adhesion.

The purpose of this work was to elucidate how controlled punch surface preparation and subsequent atmospheric aging modify the surface chemistry of the punch and, in turn, govern powder adhesion and sticking behavior. A carefully cleaned surface offers a consistent starting point for the evaluation of sticking but may differ substantially from the state of punches used for actual tableting operations. To adjust consistently the state of the punch surface after the initial cleaning, we used an aging process by exposing punches to room temperature and fixed humidity for a period of time. This gave us the opportunity to understand the evolution of clean surfaces in atmospheric conditions and led to a controllable condition that may better represent a production punch rather than the newly cleaned punch. At the same time, this work provides interesting insights into the interactions between adhering material and punch surface chemistry that lead to sticking.

## Materials and Methods

### Materials

Punches with removable, 9.525 mm-diameter, round, flat-faced punch tips (Grade S7 tool steel, Natoli Engineering) were used in these studies. Commercially available Simichrome (Happich GmbH) polishing paste was used for this study, but other alternatives may also exist. Simichrome contains 8–10 µm alumina particles suspended in a paste of protective oils. Simichrome residues can be removed by soap and water. An alkaline metal cleansing solution (MC-3, Branson Ultrasonics) diluted at 1:10 with distilled water was used.

Acetylsalicylic acid (ASA) (Sigma-Aldrich), ρ_true_ = 1.40 g/cc [31], and ibuprofen (IBU) (Spectrum Chemicals), ρ_true_ = 1.12 g/cc [32], were chosen as active materials that have been reported to exhibit sticking propensity [2–4, 17, 33, 34]. Mannitol SD200 (Roquette Pearlitol), ρ_true_ = 1.51 g/cc [35] was selected as an excipient known to exhibit a range of sticking propensities [3, 4, 17, 34]. Magnesium stearate, ρ_true_ = 1.09 g/cc [35], was used as the external lubricant. Starch 1500® (Colorcon), ρ_true_ = 1.49 g/cc [35], and microcrystalline cellulose (Avicel® PH-102), ρ_true_ = 1.56 g/cc [36], were the chosen non-sticking excipients.

**Fig. 2:**
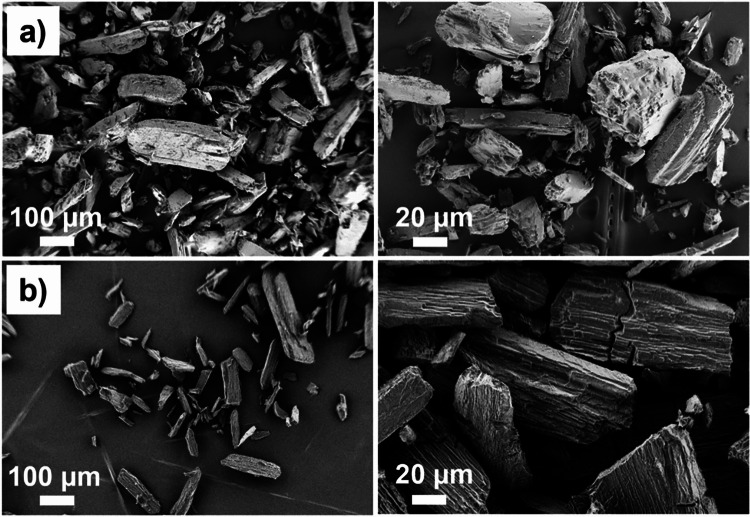
Scanning electron microscope images of as-received active pharmaceutical ingredients (**a**) acetylsalicylic acid and (**b**) ibuprofen powders prior to compaction.

**Fig. 3:**
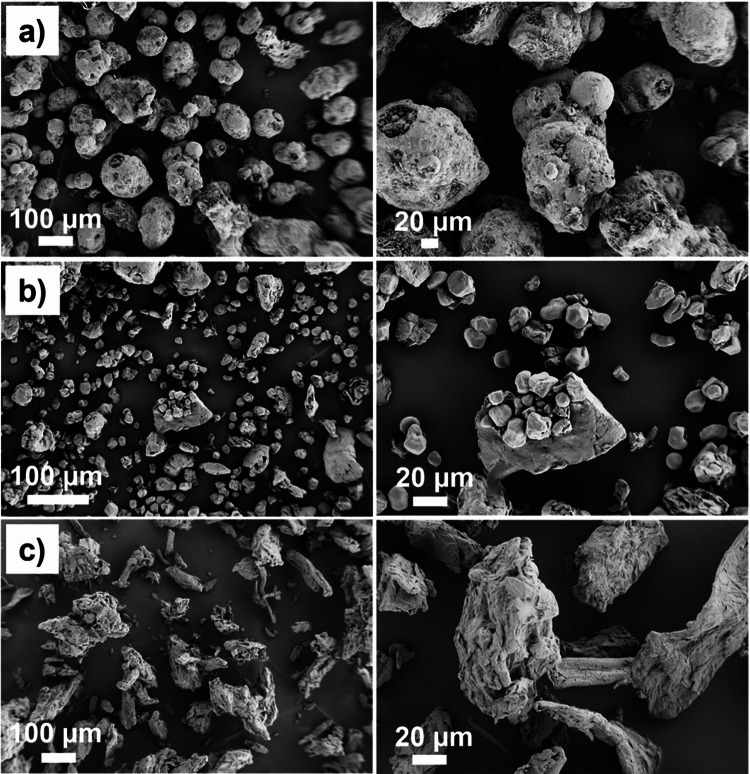
Scanning electron microscope images of as-received excipient ingredients (**a**) mannitol, (**b**) starch (Starch 1500), and (**c**) microcrystalline cellulose (PH102) powders prior to compaction.

The various materials in the as-received condition are shown in Fig. 2 and Fig. 3. Mannitol is a spray-dried powder agglomerated into spherical shapes. The starch particles also exhibit a roughly spherical shape but are slightly smaller than the mannitol particles. ASA and IBU contain mostly elongated particles. Microcrystalline cellulose particles have no sharp edges, but they are present with aspect ratios in the range of 1–5.

### Compaction and Laser Sensor

**Fig. 4:**
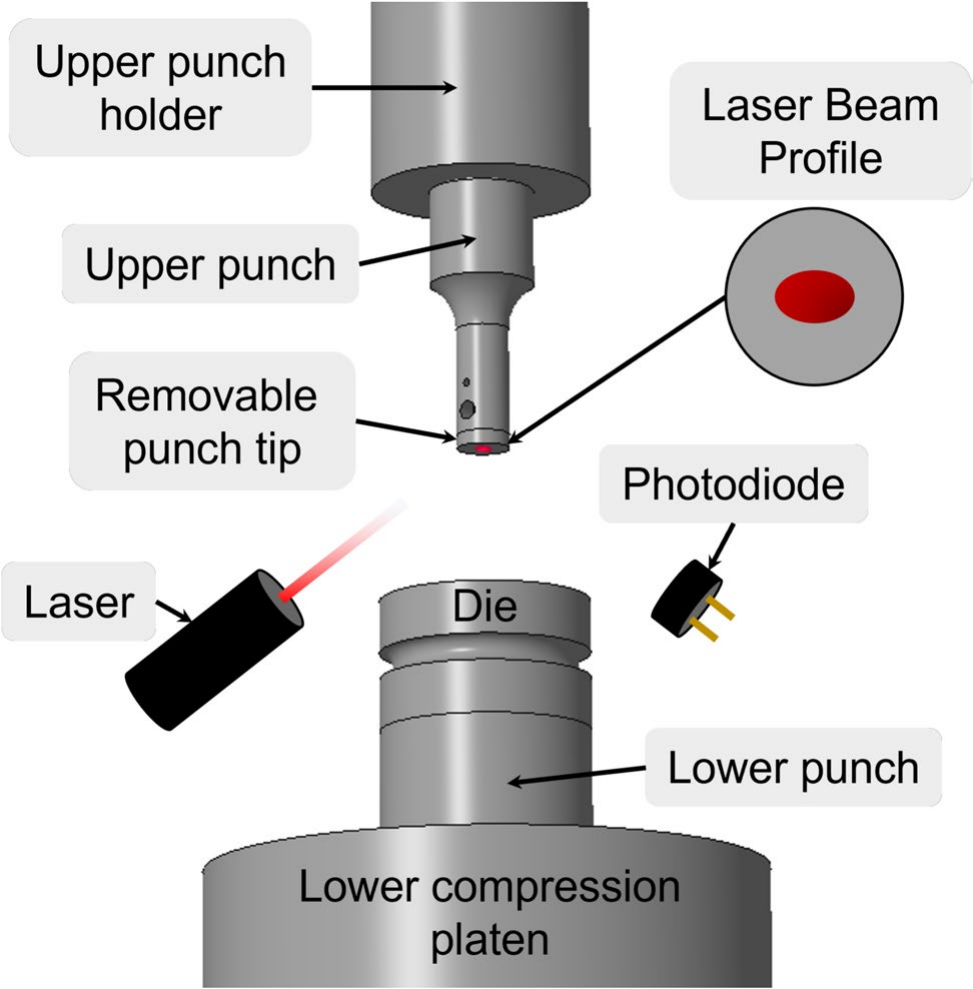
Laser sensor setup integrated with the servo hydraulic testing machine. The schematic with the ellipse within the circle indicates the size of the laser spot on a punch of 9.525 mm diameter.

A servo hydraulic testing machine (MTS Systems Corporation) was used to compress powders into tablets. The setup comprises an upper attachment for the punch and a lower punch mounted on a flat platen attached to the servo hydraulic machine, shown below in Fig. 4. A set of four custom flat removable upper punch tips (9.525 mm diameter) were used in this study. This design allowed for easy removal of the punch tip for cleaning, SEM imaging, and mass measurements.

Powder was pre-weighed for a targeted in-die relative density of 90–95% for all powders. The punch tip was attached to a custom Type B extended upper punch using a set screw. The upper punch surface was analyzed using the laser apparatus. The die and lower punch assembly was aligned with the upper punch, and the powder was compacted under displacement control. The compaction consisted of a single upper punch action following a triangular compaction profile where the loading and unloading speeds were set to 0.2 mm/s.

Tablet mass, thickness, and diameter were measured immediately after compaction. Tablets were diametrically compressed to failure at a speed of 0.5 mm/min in a CT5 tabletop mechanical testing machine (Engineering Systems NOTTM) using a 500 N load cell. The maximum load at failure was recorded, and the tensile strength of the tablets was calculated from the measured force at failure under diametral compression according to the following formula:

1 $${\sigma }_{T}=\frac{2P}{\pi Dt}$$

where *P* is the maximum load at failure, *D* is the diameter and *t* is the thickness of the tablet. The relative density is calculated using the following formula:

2 $$RD=\frac{\rho }{{\rho }_{t}}$$

where *ρ* is the apparent density of the tablet and *ρ*_*t*_ is the true density of the powder.

The compaction pressures and resulting tablet properties are summarized in Table II. All experiments targeted an in-die relative density of 90–95%.

**Table II:** Summary of Compaction Pressures, Densities, and Tensile Strengths

**Material**	**Compaction Pressure [MPa]**	True Density, [g/cc]	**Relative Density [%]**	**Tensile Strength [MPa]**
Mannitol (SD200)	168	1.51 [35]	83.3 ± 0.2	3.45 ± 0.02
Starch 1500	84	1.48 [35]	78.0 ± 0.5	0.82 ± 0.07
Acetylsalicylic Acid	56	1.40 [31]	92.4 ± 0.5	0.72 ± 0.04
Ibuprofen	56	1.12 [32]	93.2 ± 1.2	0.63 ± 0.06
Microcrystalline Cellulose (PH102)	140	1.56 [36]	86.6 ± 0.5	6.59 ± 0.06

The upper punch tip was photographed using a 15 × macro lens on a 12-megapixel Galaxy S10 + (Samsung, South Korea) and its mass was measured with an analytical balance (Mettler-Toledo, XS64, ± 0.1 mg). The upper punch tip was then cleaned as described in the Section on upper punch tip aging. The lower punch and die were prepared as described in Sect. "Lower Punch and Die Preparation", below. Four replicates were made for each experimental condition. For aged samples, the four punch tips were stored at 55% relative humidity for 1 h following the last cleaning and drying step prior to compaction, details provided in Sect. "Upper Punch Tip Aging". This procedure was repeated for punch tip storage times of 10 min, 1, 4, 24, and 72 h.

A laser apparatus following the design of our earlier work [2] was constructed to fit onto the servo hydraulic mechanical testing machine (MTS Corporation), Fig. 4. The laser angle was set to approximately 47° and the beam exhibited an elliptical spot size (4.3 mm by 3 mm). The light intensity reflected from the upper punch tip was measured before and after making a compact. The fraction of light that is not reflected (I_loss_) is reported as a percentage relative to the clean punch reflection:

3 $${I}_{loss}(\%)=\left(1-\frac{{I}_{meas}}{{I}_{max}}\right)\times 100$$

where *I*_*meas*_ is the measured intensity after compaction and *I*_*max*_ is the maximum intensity measured on a clean punch tip prior to compaction. Intensity loss is used as a measure to detect the presence of adhered material and indirectly to quantify area coverage on the punch tip during each compaction. An average of the intensity values is reported.

### Upper Punch Tip Surface Preparation

Before testing each new material, the punch tips were hand-polished with Micro-Mesh® sandpaper ranging from 6,000 to 12,000 grit, for at least one minute per grit size. Between polishing steps, the punch tips were rinsed with 70% isopropyl alcohol and wiped with a Kimwipe (Kimtech Science). Prior to a study, each punch tip was polished using Simichrome paste and a cotton swab with excess paste removed using a Kimwipe. This process effectively removes a thin layer from the metal surface of the punch.

The punch tip was submerged in 4 mL of a 10% aqueous alkaline cleaning solution and sonicated for 5 min. The punch tip was removed with forceps and rinsed using a stream of distilled water, then the wetted tip was dried for up to 10 s under the flow of argon. The tip was then submerged into a 4 mL vial containing deionized water and this vial was placed in an ultrasonic bath for 5 min followed by a 10 s drying under argon gas. The deionized water rinsing step was repeated twice as recommended in [23, 25]. At the final drying step, the punch tip was used within one minute of drying (denoted as ‘0 h’ or ‘immediate’).

**Fig. 5:**
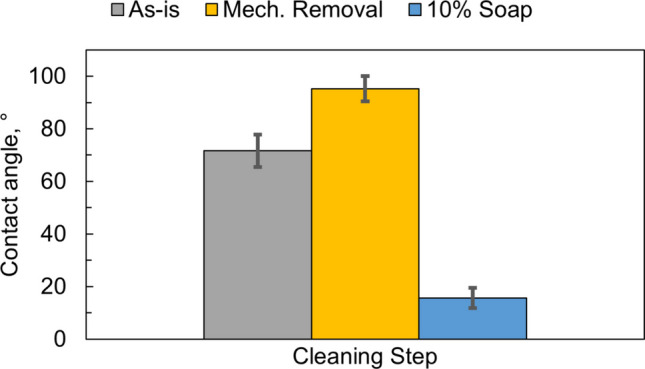
Contact angle of the punch surface before cleaning, after polishing and after alkaline cleaning, rinsing and drying.

To verify the cleaning procedure, a set of stored punch tips (hereafter referred to as ‘as-is’) was examined for wettability before the cleaning procedure and during the cleaning procedure. They were stored in the laboratory for approximately four months after the tooling had been wiped with a Kimwipe soaked in 70% isopropyl alcohol, dried with a Kimwipe, and left uncovered without any protective oil. The contact angle of the surface of these tips as retrieved from storage was 72± 6°. Immediately after polishing, the contact angle increased to 95° ± 5, Fig. 5, due to residual kerosene present in the polishing paste [37]. Following cleaning with the MC-3 solution and rinsing with deionized water, the contact angle decreased to 16° ± 4. The low contact angle after rinsing and drying demonstrates that the oils from the polishing paste were effectively removed, but it does not guarantee by itself a clean surface. The rinsing and drying process should also remove effectively any MC-3 traces. The data presented later in the Section on surface analysis using XPS clearly show that no residue from MC-3 remained on the as cleaned surface.

To quantify repeatability, 23 total contact angle measurements were completed by two users following the suggested cleaning procedure and measured 16° ± 4. This demonstrates consistency in the surface preparation following the suggested method.

### Upper Punch Tip Aging

To evaluate the effects of aging, each punch tip was first prepared according to the cleaning procedure described in Sect. "Upper Punch Tip Surface Preparation". After the final drying step, the punch tip was stored in a container with a saturated sodium bromide salt solution (55% RH) at room temperature for 10 min, 1, 4, 24, and 72 h.

### Lower Punch and Die Preparation

For compaction, the die and lower punches were cleaned using a Kimwipe soaked in 70% isopropyl alcohol and dried under ambient conditions. The die wall and lower punches were lightly dusted with magnesium stearate using a cotton swab, and any loose powder was removed with a Kimwipe, so that no visible residue remained on the punch or inside the die. This step was performed to prevent ejection problems after compaction.

### Wettability

A practical quality control method is essential when evaluating surface preparation consistency. A detailed study of surface chemistry by X-ray photoelectron spectroscopy (XPS) is best in terms of accuracy but is time consuming and requires access to specialty instrumentation. Wettability measurements can serve as a rapid alternative to XPS and have been shown to yield consistent values [38]. Standardization of the post-cleaning time and other testing conditions is essential for reliable results. Although the contact angle does not directly correspond to a unique ‘surface state’, being influenced by roughness, porosity, and residue type (e.g., hydrophobic oils vs. hydrophilic surfactants or salts), changes in contact angle can indicate variations in the cleaning process.

Static contact angles were estimated using the sessile-drop technique. Measurements were taken at ambient conditions during the surface preparation and aging process of the punch tip surfaces. The measured values provided a rapid indication of surface changes, serving as a substitute for the more accurate but time consuming XPS analysis. Measurements were taken on a 20 μL drop of liquid carefully dispensed on the punch tip. Images of the droplet were captured with a 15 × macro lens on a digital camera. The images were processed in ImageJ using the angle tool.

### Determination of Surface Energies

The Owens–Wendt–Rabel–Kaelble (OWRK) method was applied to the contact angle data for the test liquids to evaluate surface energy ($\gamma$) parameters, namely the polar ${(\gamma }^{p})$ and dispersive (${\gamma }^{d})$ components. Distilled water and diiodomethane were chosen as the test liquids due to their dominant polar and dispersive components, respectively. The OWRK theory was developed for a two-component model to separate the interfacial tension according to interactions between the molecules, specifically the polar and dispersive interactions. Within the OWRK framework, polar adhesive forces arise due to permanent dipole–dipole interactions or Keesom forces that only exist in polar molecules. Dispersive (London) forces are weaker and originate from temporary or induced dipole interactions.

Wettability measurements were performed on the punch tip using both test liquids. The punch tip was prepared following the cleaning procedure (‘immediate’ condition) and then aged for 1, 24, and 72 h. Surface energy was calculated from the contact angle data using the OWRK method, which requires two liquids with known total surface tension and known polar and dispersive components (listed in Table III). Three replicate measurements were taken at each time point. The OWRK analysis and calculations follow ASTM D7490 [39] and are based on the following equation:

4 $$\frac{1}{2} {\gamma }_{l}\left(1+\mathit{cos}\theta \right)=\left[{\left({\gamma }_{l}^{d}{\gamma }_{s}^{d}\right)}^{1/2}{+\left({\gamma }_{l}^{p}{\gamma }_{s}^{p}\right)}^{1/2}\right]$$

where *θ* is the average contact angle for the test liquid on the test specimen, ${\gamma }_{l}$ is the surface tension of the test liquid, ${\gamma }^{d}$ is the dispersion component of the liquid and the solid, and ${\gamma }^{p}$ is the polar component of the liquid and the solid.

**Table III:** Surface Energy Data of the Test Liquids Used; Values from ASTM Standards (39)

	**Surface Energy** $[{\boldsymbol{m}}{\boldsymbol{J}}/{{\boldsymbol{m}}}^{2}]$		
Test Liquid	${{\boldsymbol{\gamma}}}_{{\boldsymbol{l}}}$	${{\boldsymbol{\gamma}}}_{l}^{{\boldsymbol{p}}}$	${{\boldsymbol{\gamma}}}_{l}^{{\boldsymbol{d}}}$
Distilled Water	72.8	51.0	21.8
Diiodomethane	50.8	1.3	49.5

### Scanning Electron Microscopy

The punch tips and powders were examined using a scanning electron microscope (Zeiss Supra 50VP FESEM) to document the morphology of the starting materials and the adhered material after compaction. The punch surfaces and powders were examined at a low accelerating voltage (1 kV) under high vacuum without a conductive coating, except for IBU, which degraded upon exposure to 1 kV. The powders were placed on carbon-adhesive tape prior to examination. High and low magnification images of the punch tips were analyzed following the procedure described by Tsosie *et al.* [18].

### X-ray Photoelectron Spectroscopy

X-ray photoelectron spectroscopy (XPS; Physical Electronics VersaProbe) was performed in an ultrahigh vacuum chamber (∼1 × 10⁻^7^ Torr) equipped with a monochromatic Al Kα (1486.7 eV) X-ray source operated at 15 kV and 50 W. The take-off angle was set to 45°. A pass energy of 117.4 eV was used for wide range spectra (survey) and 23.5 eV for high-resolution spectra. Quantitative analysis was completed using CasaXPS (Casa Software Ltd.) processing software using a Shirley background and a Gaussian-Lorentz line shape. For depth profiling by etching, argon ions were used at times of 1 to 5 min. All binding energies were referenced to the carbon C-H photopeak at 285.0 eV.

## Results

### Sticking Behavior

**Fig. 6:**
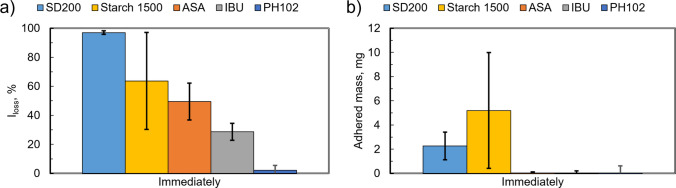
Results of all materials after initial surface preparation (immediately): (**a**) laser measurements and (**b**) corresponding mass measurements. Error bars indicate the standard deviation of experiments.

**Fig. 7:**
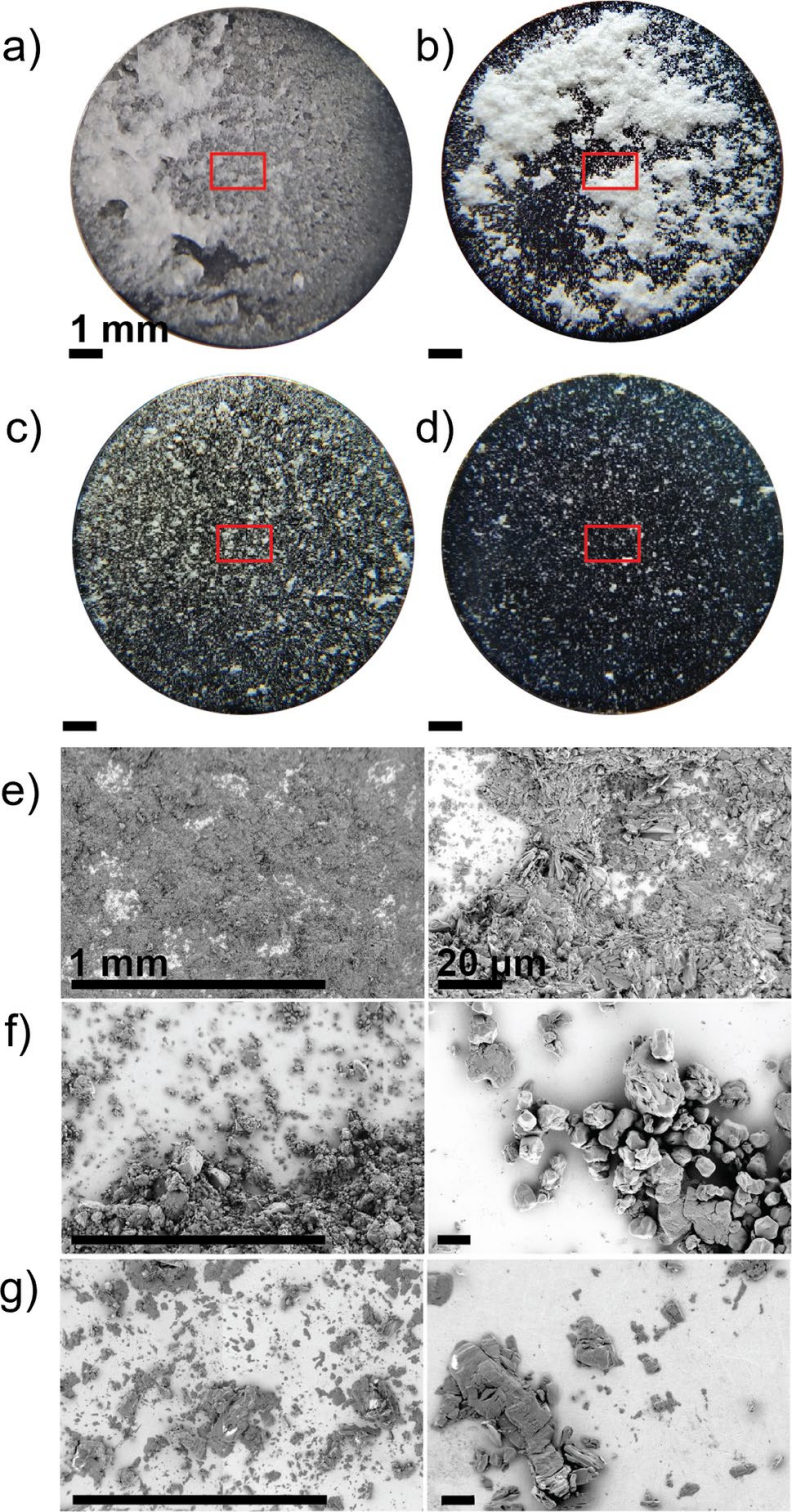
Optical (**a**-**d**) and scanning electron microscopic (**e**–**f**) observations of all materials on a punch following the cleaning procedure. Red box indicates approximate region examined by laser apparatus as well as the scanning electron microscope. (**a**) & (**e**) Mannitol, (**b**) & (**f**) starch, (**c**) & (**g**) acetylsalicylic acid and (**d**) ibuprofen. Ibuprofen was not imaged in the scanning electron microscope as it evolved significantly under vacuum. The magnification bar is the same for each column of scanning electron microscope photos.

Punch tips cleaned according to the procedure described above were tested for sticking with four different materials. The results of the laser sensor and mass measurements are shown in Fig. 6. Moderate sticking was observed for ASA and IBU with 50% and 29% punch tip coverage, respectively, with the adhered mass below the sensitivity of the analytical balance (Mettler-Toledo, XS64, ± 0.1 mg). Compared with our prior work [18, 40], where the tip was just wiped with IPA more sticking was observed on the tip surface prepared based on the procedure outlined in Sect. "Upper Punch Tip Surface Preparation". The morphology of the sticking layer for ASA, Fig. 7, consisted of a mixture of fragments and highly deformed particles consistent with prior observations [18]. ASA and IBU display similar coverage across the diameter with a mostly speckled appearance that includes a variation of particle sizes. The majority of the adhered ASA particles are less than 10 μm. There are some larger particles, 40 μm (minor axis) and 80 μm (major axis), present that appear to be more heavily deformed (thinner platelets) when compared to the starting material. These observations are consistent with the types described in our previous work [18].

Mannitol exhibited substantial sticking with approximately 97% of the punch surface covered by adhered material. Approximately 2 mg of material was deposited making the equivalent thickness of the sticking layer to be ~ 10 μm. The adhered material does not retain much of the spherical/agglomerate spray-dried shape (Fig. 3) but rather appears crushed. Visually, the adhered material appears to consist of remnants of the needle-like particles that were agglomerated during spray-drying, as shown in the SEM images in Fig. 7. Other grades of mannitol have been reported to show some sticking propensity with approximately 5–90 μg after a single compaction [4, 12].

The sticking exhibited by starch was unexpected. Waimer, et. al. [17] reported no sticking for the same grade of starch but the exact cleaning procedure was not described. In addition, the punch was covered by a sheet metal that could have different characteristics from a solid machined punch. To the best of our knowledge there are no studies indicating that Starch 1500 sticks to punches. Another study explicitly reported that starch does not stick [41]) but, as in other cases, it did not specify the punch preparation procedure used in the experiments. It is therefore reasonable to infer that starch sticking was induced by enhanced adhesion to the freshly cleaned S7 tool steel punch surface. A 64% coverage was observed with approximately 5 mg of adhered starch corresponding to a rather large thickness of the order of 50 µm. SEM observations of the adhered material appear more as large groups of particles throughout the surface of the punch, and this is most likely the reason for the large variation in both laser sensor and mass results. The largest contribution of adhered particles comes from partially flattened agglomerates. There is also a mixture of deformed and non-deformed single particles remaining on the surface.

### Surface Analysis Using XPS

**Fig. 8:**
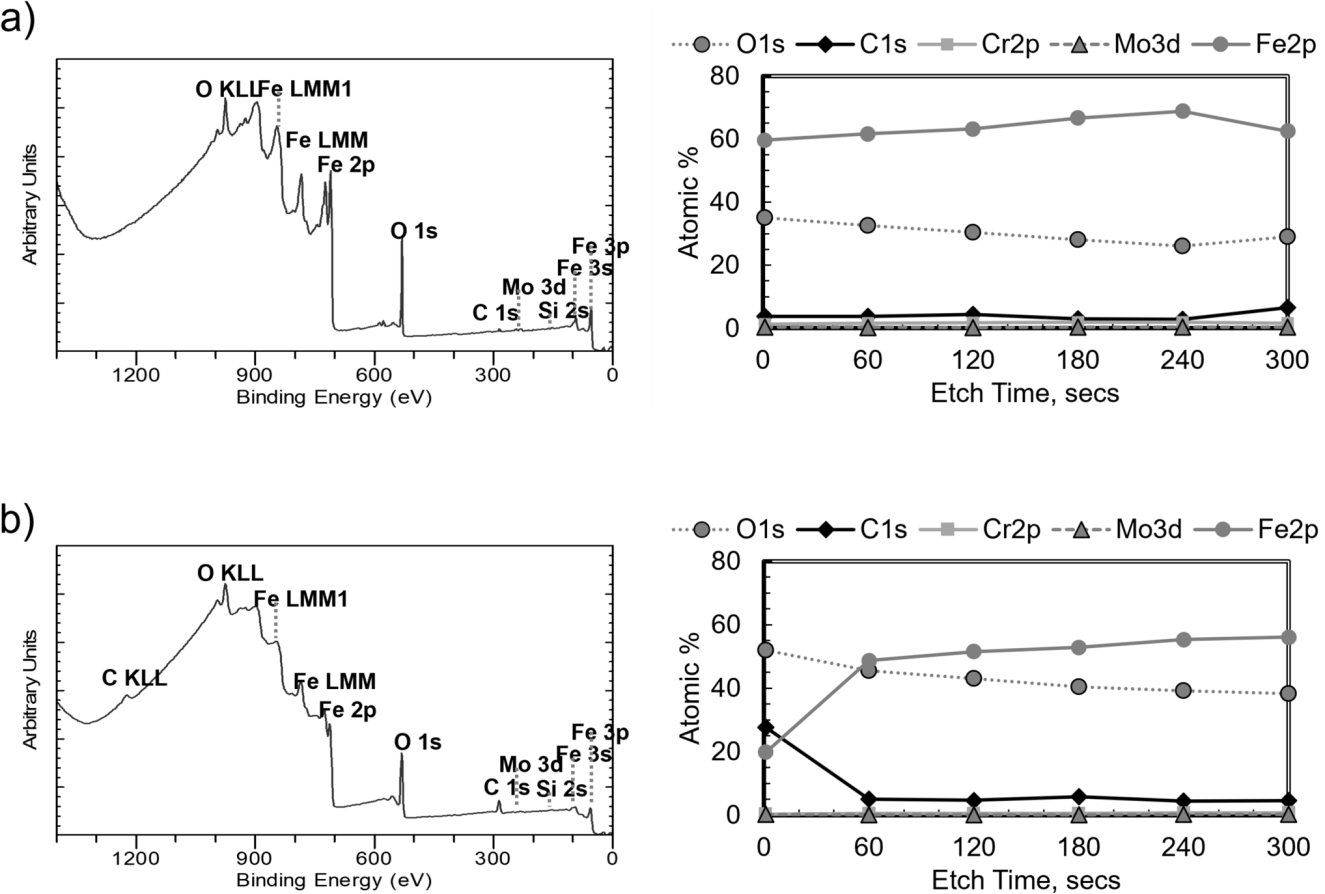
Survey scans (left) and depth profiling of the punch tips showing descriptive elemental compositions (right) following the (**a**) cleaning procedure and (**b**) 72-h in storage at 55% RH.

XPS was used to characterize the chemical state of the S7 tool steel punch surface immediately after cleaning and after controlled atmospheric aging. The survey spectra of the as-cleaned surface (t ~ 0 h) showed a thin native film consisting primarily of iron oxide/oxyhydroxide, with small amounts of Cr(III) and Mo oxides, and capped by a very thin adventitious-carbon layer (C 1 s at ~ 5 at%), Fig. 8a. This film forms rapidly during the short interval between cleaning and loading into the XPS chamber. Importantly, no peaks associated with the MC-3 alkaline cleaner were detected. MC-3 contains sodium metasilicate and an ethoxylated nonylphenol surfactant [42], which would produce Na 1 s (~ 1071 eV), Si 2p (~ 102–103 eV), and strong C–O (~ 286.3 eV) contributions in the C 1 s region. The absence of these signals confirms complete removal of cleaner residues.

After storage for 72 h at 55% RH, the survey spectra changed. The Fe 2p peak decreased in intensity (from ~ 60% to ~ 20% of the total signal), while O 1 s and C 1 s increased (from ~ 35% to ~ 55% and from ~ 5% to ~ 30%, respectively), Fig. 8b. These changes indicate growth of a thicker oxide/hydroxide layer and further accumulation of adventitious carbon during atmospheric exposure. The aged surface also exhibited more intense carbon Auger features and a stronger C 1 s peak at ~ 284.5 eV, confirming carbonaceous contamination typical of metals stored in air.

**Fig. 9:**
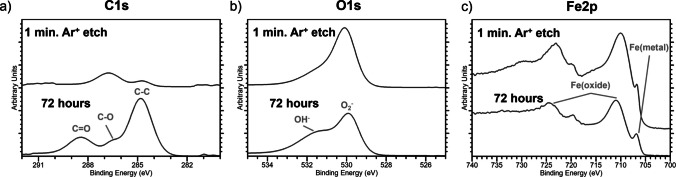
High resolution spectra for (**a**) C1s, (**b**) O1s, and (**c**) Fe2p following storage at 55% RH for 72 h and after a 1- min Ar + etch.

High-resolution spectra provided additional insight beyond the survey scans. The C 1 s region revealed distinct C–C, C–O, and C = O bonding environments, while the O 1 s region showed both oxide and hydroxide components, Fig. 9. These chemical states cannot be resolved from the survey spectra alone and reflect the chemical complexity of the surface film with aging.

Depth profiling using Ar⁺ sputtering further clarified the structure of the surface layers. A 1-min etch substantially reduced the carbon signal (from 28 at% to 5 at%) and collapsed the OH/H₂O shoulder in the O 1 s region, indicating removal of most of the adventitious-carbon layer and reduction of hydroxide species, Fig. 9. Fe 2p intensity increased correspondingly as the underlying oxide was exposed. Longer etching times continued to decrease the O 1 s signal but did not eliminate oxygen entirely, consistent with the persistence of alloy oxides (e.g., Cr₂O₃, SiO₂) and with sputter-induced mixing that distributes oxygen deeper into the near-surface region.

Together, these XPS results show that the punch surface evolves substantially under atmospheric exposure, transitioning from a high-energy, thin-oxide surface immediately after cleaning to a lower-energy surface covered by a thicker carbon/oxide film. The growth of this nanometer-scale layer is consistent with trends observed in steel and other metals [22, 25, 43] and provides a basis for understanding the time-dependent changes in wettability and sticking behavior discussed in later sections.

### Surface Aging and Wettability Evolution

Although XPS provides the most accurate surface characterization, wettability is often correlated with the kinetics of surface evolution (see e.g., [44]). In this section we use wettability to follow the changes of the surface during cleaning as well as aging of the cleaned punches during their exposure in air at 55% RH.

The results in Section on Sticking Behavior showed that a freshly prepared punch surface exhibited aggressive sticking behavior even for materials that in general are considered non sticking (e.g., starch). Therefore, a transition appears to occur as the initially cleaned punch surface gradually changes during atmospheric exposure. To study the kinetics of surface evolution and its effect on sticking propensity, a set of as-cleaned punch tips was progressively exposed to constant 55% RH and room temperature for up to 72 h.

**Fig. 10:**
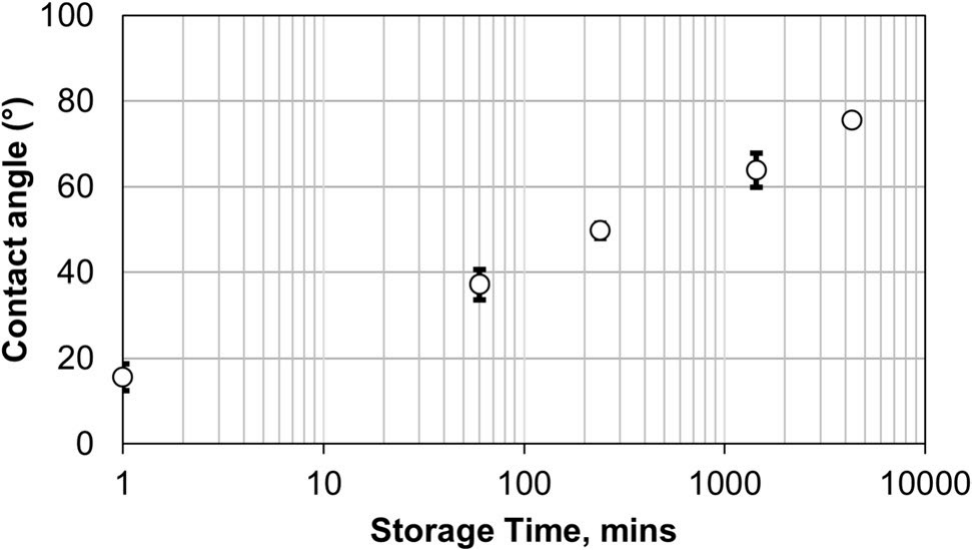
Wettability measurements of punch tips stored for ‘x’ hours under 55% RH conditions. Error bars indicate the standard deviation of experiments.

During this period, the contact angle increased from approximately 16° for the as-cleaned surface to 76° after 72 h (Fig. 10), which is slightly lower than the 85 ± 3.2° measured for the as-stored surface. This transition reflects increasing surface contamination resulting from interaction between the metal surface and the atmosphere, consistent with observations on stainless steel, where the static contact angle reached ~ 80° after 2–3 days of exposure [28].

The contact angle measurements for the punch tips following the cleaning and aging procedures are summarized in Table IV. The contact angle measured with distilled water increased from 16° to 76° after 72 h of storage, whereas the angle measured with diiodomethane increased from 21° to 31° after 1 h and remained nearly constant (~ 40°) thereafter. The total surface energy decreased with storage time, primarily due to a reduction in the polar component during aging. The corresponding calculated surface energies are given in Table V. The total surface energy decreased following storage, with the polar component showing the largest decrease over the aging process. The dispersive component remained nearly constant, measuring between 34 and 36 mJ m⁻^2^ throughout the aging process, indicating little change with storage.

**Table IV:** Measured Contact Angle of the Tested Punch Surfaces. Results are the Arithmetic Mean of Three Replicates of Contact Angle Measurements of the Two Test Liquids

	**Contact Angle** $[^\circ ]$			
Liquid	Immediately	1 h	24 h	72 h
Distilled Water	16 ± 3.1	37 ± 3.5	64 ± 4.0	76 ± 1.3
Diiodomethane	21 ± 4.0	31 ± 5.0	39 ± 5.0	40 ± 3.0

**Table V:** Measured Surface Free Energy of the Tested Punch Surfaces

	Surface Energy*$[{\boldsymbol{m}}{\boldsymbol{J}}/{{\boldsymbol{m}}}^{2}]$		
Time	${{\boldsymbol{\gamma}}}_{{\boldsymbol{S}}}^{{\boldsymbol{p}}}$	${{\boldsymbol{\gamma}}}_{{\boldsymbol{S}}}^{{\boldsymbol{d}}}$	${{\boldsymbol{\gamma}}}_{{\boldsymbol{S}}}$
Immediately	36.9	36.0	72.9
1 h	28.5	34.1	62.6
24 h	12.3	34.2	46.5
72 h	5.8	36.0	41.8

^*^Results are taken as an average of three replicates of contact angle measurements using two test liquids. ${{\boldsymbol{\gamma}}}_{{\boldsymbol{S}}}$ – total surface free energy**,** ${{\boldsymbol{\gamma}}}_{{\boldsymbol{S}}}^{{\boldsymbol{p}}}$ – polar component, ${{\boldsymbol{\gamma}}}_{{\boldsymbol{S}}}^{{\boldsymbol{d}}}$—dispersive component

### Sticking Behavior Following Storage

**Fig. 11:**
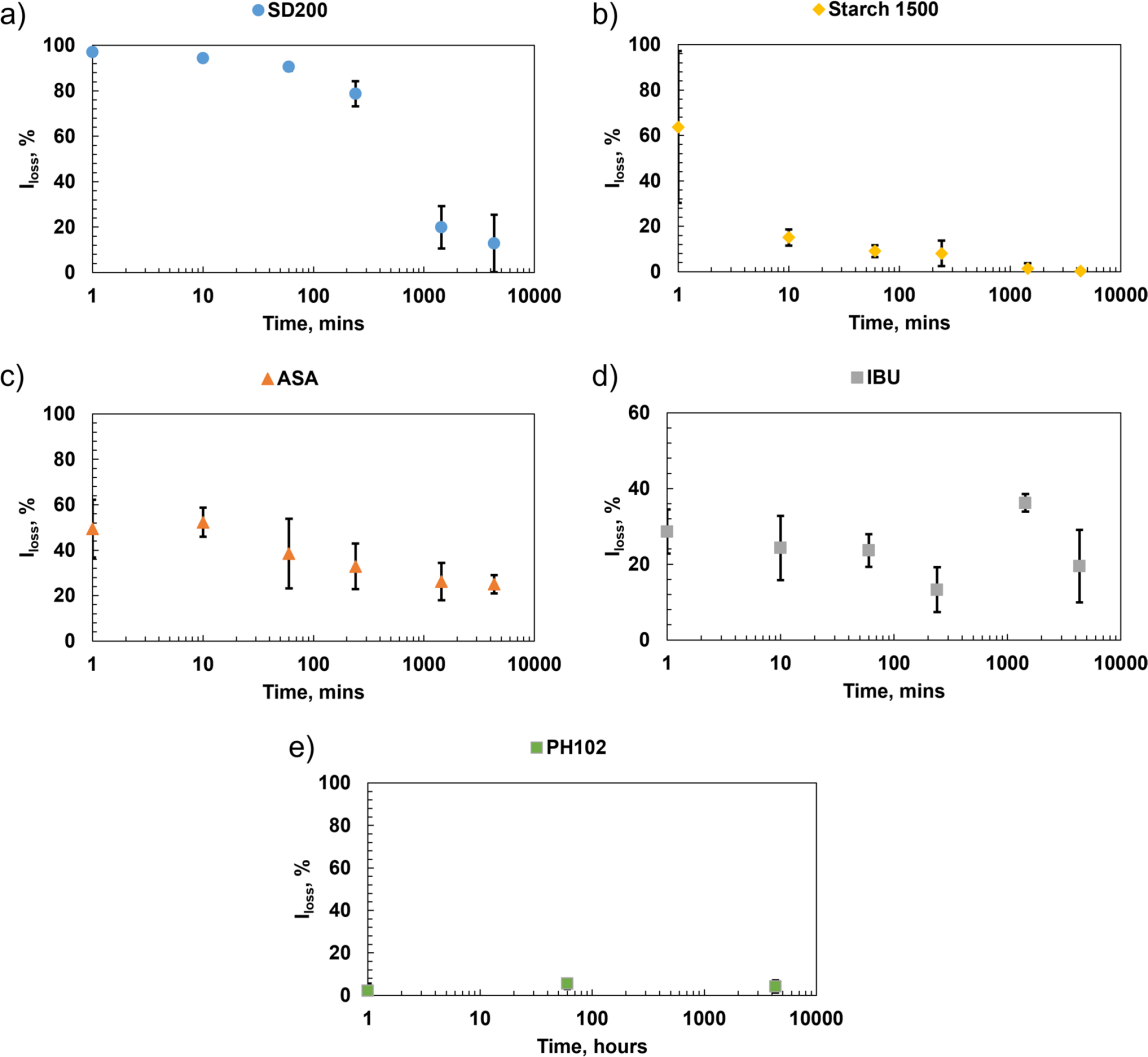
Laser reflection intensity loss *versus* aging time: (**a**) mannitol, (**b**) starch, (**c**) acetylsalicylic acid, (**d**) ibuprofen and (**e**) microcrystalline cellulose. Error bars indicate the standard deviation of experiments.

Figure 11 presents the laser sensor measurements, expressed as the percentage of light not reflected from the surface. This parameter scales with the coverage of area by adhered material [2]. For all materials except ibuprofen, a systematic reduction of the area covered (less sticking) is observed with increasing time of storage of the punch tips.

**Fig. 12:**
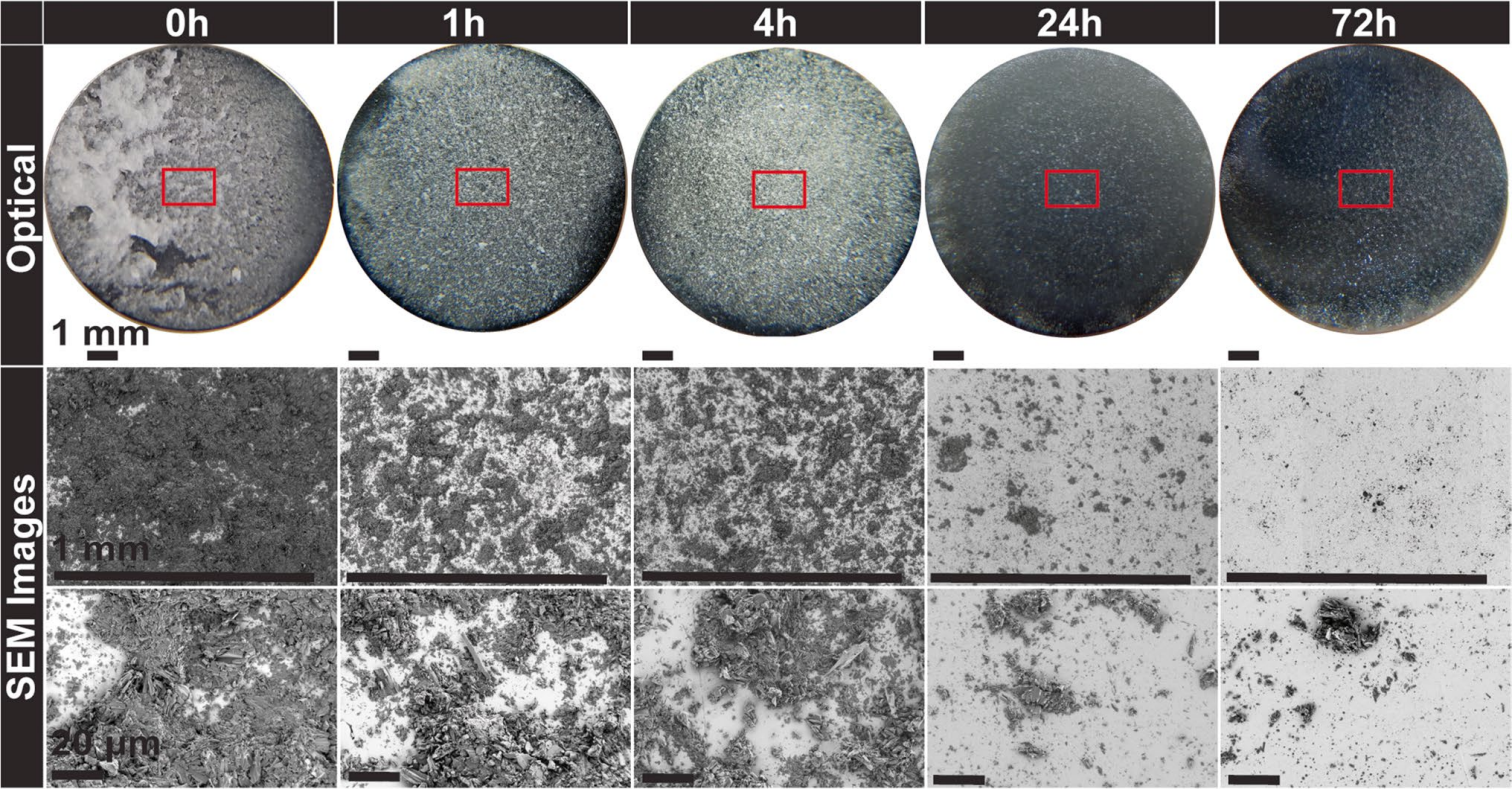
Optical and scanning electron microscope observations of mannitol. The boxes indicate the approximate regions examined by both the laser apparatus and the scanning electron microscope.

Mannitol is affected the most by the changes induced to the punch tip surface due to aging. A significant decrease in sticking with time was observed, see Fig. 11a for laser sensor results and Fig. 12 for visual appearance. At 0 h, the separation of adhered material is through the top portion of the tablet, consisting of groups of crushed particles spread across the punch tip. Its behavior differs from the other materials in that it appears to undergo an evolutionary stage before a larger drop in sticking occurred after 4 h of storage. The punch tip after 1 h of storage shows a mixture of similar groups of crushed particles that are within the 50 μm range of the starting material. In addition, there are smaller agglomerates present that appear to be the needles formed inside each particle during spray drying. The appearance after 4 h of storage was very similar to the SEM images after 1 h. After 24 h of storage, the amount of adhered material was substantially reduced, and most remaining particles were much smaller (< 20 µm) than the starting material. After 72 h of storage, the appearance is very similar to 24 h with even less adhered material. The majority of the adhered particles are less than 5 μm. For all compacts, tablet strength remained constant; only the interaction between mannitol particles and the punch surface changed.

**Fig. 13:**
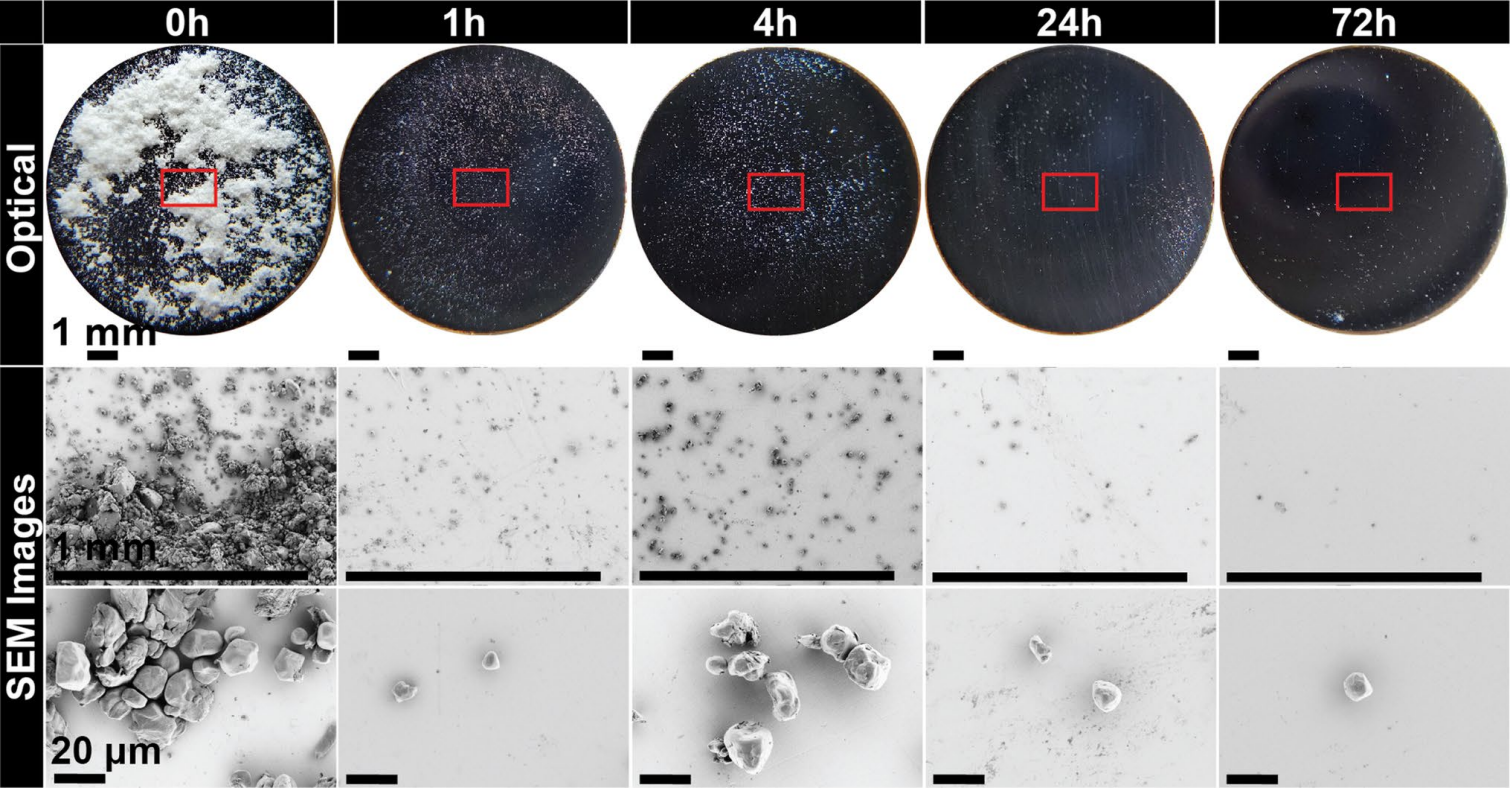
Optical and scanning electron microscope observations of Starch 1500. Red box depicts the approximate area examined in scanning electron microscope at higher magnifications.

Starch also showed unexpected sticking when tested immediately after cleaning, despite being typically reported as non sticking material. Sticking was substantially reduced within one hour of storage and almost completely disappeared after 24 h, Fig. 11b. The optical observations depict the same area coverage detected by the laser with no differences across the diameter after 1 h, Fig. 13. The SEM examinations show a clear difference in appearance after 1 h of storage compared to 0 h, Fig. 13. After 1 h of storage, there is significantly less material, with many of the remaining particles appearing to be non-deformed single particles (< 20 μm) rather than groups of particles. This is also similar for all other storage times and even less after 24 h of storage. The particles also appear to be loosely adhered, so it is possible that they could be easily removed by air.

**Fig. 14:**
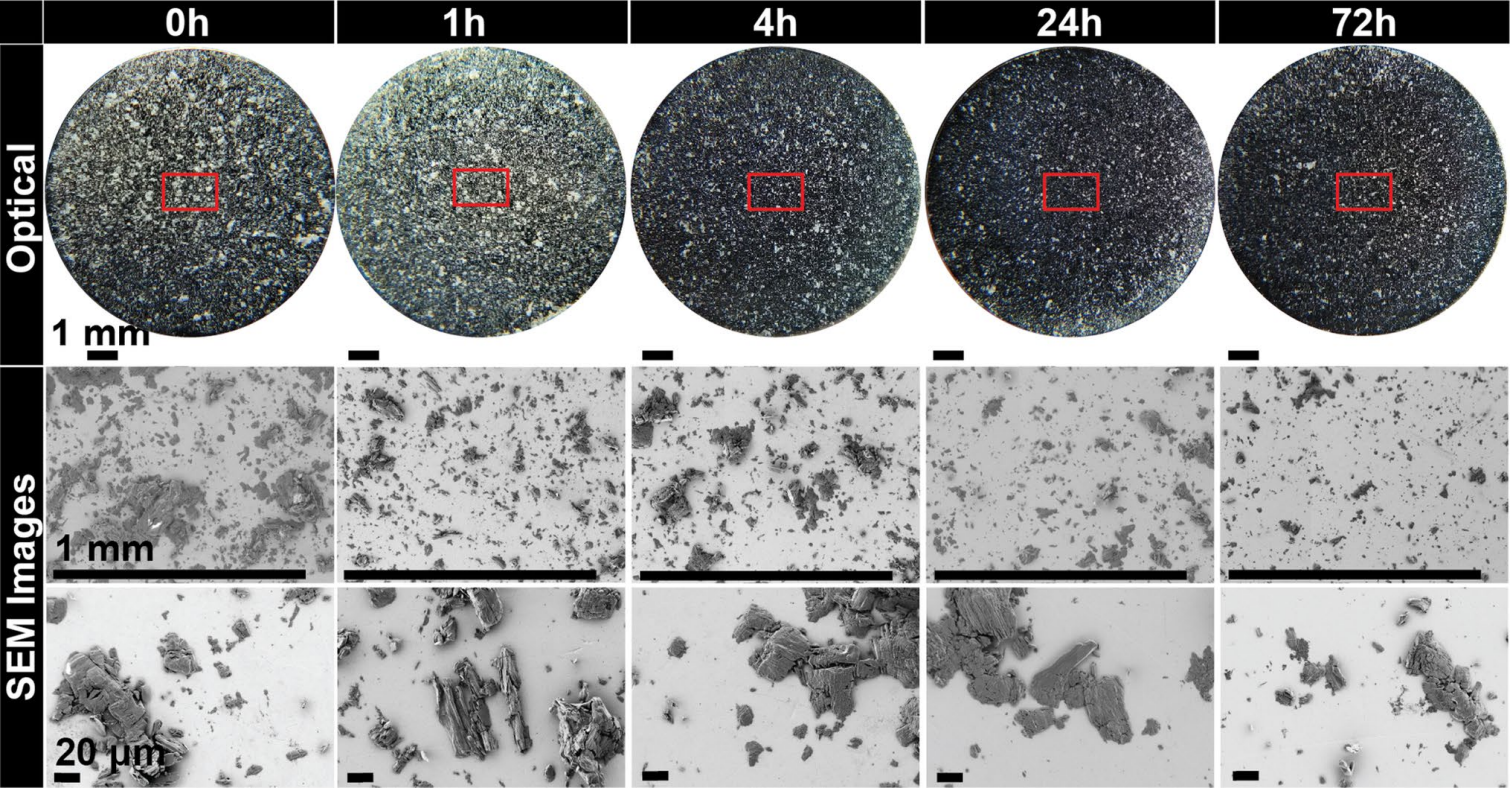
Optical and scanning electron microscope observations of acetylsalicylic acid. Red box depicts the approximate area examined using the laser apparatus as well as scanning electron microscope at higher magnifications.

ASA showed a gradual decrease with time and exhibited considerably less sticking than mannitol or starch at t ~ 0 h, Fig. 11c. The optical observations show the white specks across the diameter, Fig. 14. SEM observations of 0–4 h depict a mixture of large deformed whole particles, sharp elongated particles and fragments of particles, Fig. 14, similar to our previous observations of ASA [18]. The adhered particles are classified as Type I (fragments), Type II (particles that have a combination of deformation and fragmentation), and Type III (substantially deformed particles) consistent with the classification introduced in [18]. The larger particles are within the range of 20 μm (minor axis) by 80 μm (major axis) along with smaller particles less than 20 μm. There are noticeably fewer particles as the punch tips are aged for 4 h with even less appearance of the sharp elongated particles (Type I). After 24 h, the majority of adhered particles appear to be Type II and III particles. There are noticeably fewer particles of the 20 μm by 80 μm range and smaller particles that are < 10 μm. This is also similarly depicted at 72 h.

**Fig. 15:**
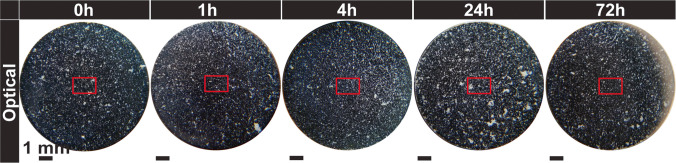
Optical observations of ibuprofen (from left to right): immediately, after 1 h, after 4 h, after 24 h and after 72 h. Red box depicts the approximate area examined using the laser apparatus.

Ibuprofen showed the least dependence on punch tip storage among the four materials, averaging about 40% surface coverage, see Fig. 11d. The optical observations also show no noticeable differences with storage time, Fig. 15, with the majority of particles appearing as white specks across the diameter. In previous observations completed by James Thomas [40], ibuprofen particles consisted of needlelike partially deformed and heavily deformed particles in the 10 µm (minor axis) by 50 µm (major axis). The adhered material is most likely a combination of fragments and agglomerates of the material.

## Discussion

While often overlooked, changes in punch surface preparation introduce significant variations of sticking behavior, making results (especially those from material-sparing experiments where only a few compactions are performed) difficult to interpret or reproduce. Most prior work on sticking, including our own, does not quantify or control the chemical state of the punch surface, even though adhesion arises directly from interactions at that interface. Our results show that surface condition immediately after cleaning and subsequent atmospheric aging, is a decisive and previously underappreciated determinant of sticking behavior. These findings indicate that reproducible sticking assessments require explicit control of surface preparation and the time elapsed between cleaning and compaction.

The cleaning procedure used here intentionally removes the prior surface layer, producing a high energy metal surface containing a very thin native oxide and minimal carbonaceous contamination. This surface is chemically distinct from punch surfaces after exposure to the atmosphere. XPS confirmed rapid adsorption of adventitious carbon, which is an organic-rich film that forms spontaneously on metals exposed to air. The formation of this film leads to a progressive decrease in polar surface energy and a corresponding increase in hydrophobicity. Wettability measurements provided a rapid and practical proxy for this evolution, showing a rapid increase in contact angle with exposure to the atmosphere.

These surface changes produced pronounced differences in sticking behavior across the tested materials. Mannitol and Starch 1500 exhibited extensive sticking to the freshly cleaned high-energy surface. Given the sizable difference in their strengths (see Table II), the sticking of Starch 1500 appears to be aided by its low mechanical strength. The high sticking tendency of mannitol on the as-cleaned surface reflects an inherently stronger adhesion given the higher strength of its tablets. It is interesting to note that there are fundamental differences in their surface chemistry. Mannitol is crystalline and exposes readily accessible hydroxyl groups capable of forming strong hydrogen bonds with metal oxide sites. As a result, even the earliest growth of the adventitious-carbon layer is sufficient to suppress adhesion almost immediately. These mechanistic distinctions explain why starch shows an early sticking tendency that disappears within minutes, whereas mannitol maintains strong adhesion until more extensive surface aging and thus a thicker adventitious carbon layer has formed.

The two APIs, acetylsalicylic acid and ibuprofen, behaved differently than mannitol and starch. Both displayed persistent sticking even after significant surface aging, and the reduction in adhesion was far less pronounced than for the two excipients. This suggests that their interactions with S7 tool steel rely less on hydrogen bonding to metal-oxide sites and more on dispersive interactions or aromatic surface contacts that remain effective even when the surface is covered by a carbonaceous overlayer. The aromatic rings and carboxyl groups in ASA and IBU may therefore support adhesion mechanisms that are comparatively insensitive to the loss of polar surface energy.

These observations provide insight into the variability frequently reported in sticking studies. Because the punch surface evolves measurably within minutes to hours of atmospheric exposure, unrecorded differences in cleaning-to-testing time can produce materially different outcomes, especially in studies based on a limited number of compactions. Standardizing this interval or adopting an aged surface as the baseline testing condition would reduce variability and improve reproducibility across laboratories.

The controlled cleaning procedure used here produces a surface that may be more reactive than typical production punches, which experience repeated sliding, impact, lubricant exposure, and abrasion by hard excipients. Such interactions may intermittently expose fresh metal, locally recreating conditions similar to those of the “immediately cleaned” state. Conversely, the aged state may better represent the average surface condition during continuous manufacturing. Considering both surface conditions, therefore, it provides complementary information. The as-cleaned state reveals worst-case adhesion scenarios, while the aged state reflects more typical operating conditions.

Further work is warranted to extend this framework to coated punches, where compositional gradients and surface treatments may complicate cleaning and aging behavior. Moreover, phenomena during tableting, such as tribological interactions, localized heating, and formulation-induced chemical modification should be evaluated to determine if and how they alter surface energetics under realistic tableting conditions.

Overall, this study demonstrates that punch-surface chemistry is a dynamic variable that significantly affects sticking behavior. By integrating controlled surface preparation, aging studies, and mechanistic analysis across multiple materials, we provide a reproducible and practically relevant framework for evaluating sticking risk in oral tablet formulation development.

## Conclusion

This study demonstrates that the state of the punch surface, defined by its chemistry immediately after cleaning and its subsequent evolution during atmospheric exposure, is a critical yet largely uncontrolled determinant of sticking measurements. We show that visually identical “clean” surfaces can differ substantially in surface energy, oxide composition, and carbon contamination, and these differences strongly influence adhesion behavior even within minutes of exposure.

By integrating controlled surface preparation, contact-angle measurements, and XPS analysis, we establish a reproducible framework for generating well defined surface states and for monitoring their rapid aging. This framework identifies the cleaning-to-testing interval as a key experimental parameter that must be standardized for reliable material-sparing sticking assessments.

The results also provide new mechanistic insight into why different excipients and APIs respond differently to surface aging. Mannitol and Starch 1500 show strong adhesion to freshly prepared but diverge sharply after aging due to intrinsic differences in the accessibility of their polar groups. Starch losing adhesion almost immediately, while mannitol continues to stick until more extensive aging occurs. In contrast, acetylsalicylic acid and ibuprofen mostly maintain adhesive interactions on aged, carbon and oxide-covered surfaces. This suggests that their adhesion relies on interactions that are less affected by the loss of polar surface energy during aging.

Together, these findings clarify the origin of unexplained variability frequently reported in sticking studies and offer practical guidance for improving reproducibility. The combined use of controlled cleaning, surface state characterization, and defined aging conditions enables more reliable interpretation of sticking behavior and offers a more realistic representation of punch surfaces encountered during manufacturing.

Overall, this work establishes surface state control as an essential component of sticking risk assessment and provides a mechanistic basis for designing more predictive and standardized compaction experiments in pharmaceutical development.
